# Depression, anxiety, and happiness in dog owners and potential dog owners during the COVID-19 pandemic in the United States

**DOI:** 10.1371/journal.pone.0260676

**Published:** 2021-12-15

**Authors:** Francois Martin, Katherine E. Bachert, LeAnn Snow, Hsiao-Wei Tu, Julien Belahbib, Sandra A. Lyn

**Affiliations:** Nestlé Purina Research, Saint-Louis, MO, United States of America; University of Lincoln, UNITED KINGDOM

## Abstract

Major life events, such as the COVID-19 pandemic, affect psychological and physiological health. Social support, or the lack thereof, can modulate these effects. The context of the COVID-19 pandemic offered a unique opportunity to better understand how dogs may provide social support for their owners and buffer heightened symptoms of stress, anxiety and depression and contribute to happiness during a major global crisis. Participants (768 pet dog owners and 767 potential pet dog owners) answered an online survey, including validated depression, anxiety, happiness psychometric scales, attitude to and commitment towards pet, and perceived social support. Potential pet dog owners were defined as individuals who did not own a dog at the time of the survey but would be very or extremely interested in owning one in the future. Dog owners reported having significantly more social support available to them compared to potential dog owners, and their depression scores were also lower, compared to potential dog owners. There were no differences in anxiety and happiness scores between the two groups. Dog owners had a significantly more positive attitude towards and commitment to pets. Taken together, our results suggest that dog ownership may have provided people with a stronger sense of social support, which in turn may have helped buffer some of the negative psychological impacts caused by the COVID-19 pandemic.

## Introduction

Worldwide, the socio-economic impacts of the COVID-19 pandemic have been extensive. Governments issued quarantine and social distancing policies as well as lockdown measures to mitigate the transmission of the disease. In the United States, there was no unified or enforceable federal response, but the federal government issued a series of public health recommendations aimed at preventing the transmission of SARS-CoV-2 (e.g., social distancing, masks wearing, temporary voluntary national lockdown in the spring of 2020). Unemployment and decreased consumer spending contributed to economic recessions. Such socio-economic factors have led to higher levels of loneliness and social impairment [1, 2]. The prolonged and significant disruption in the daily lives of people caused by the pandemic increased stress, anxiety, loneliness, and depression levels in many [3–19].

Major life events, such as the COVID-19 pandemic, affect psychological and physiological health. Social support, or the lack thereof, can modulate these effects. Social support is defined as the outcome of one or more of the following: 1) the feeling of being cared for; 2) the belief that one is loved, esteemed, and valued, and 3) the sense of belonging to a reciprocal network [20, 21]. Within this framework resides the concept that social support provides protection from pathological states and accelerates recovery from illness by acting as a buffer in times of crisis [22, 23]. Empirical studies that explored people’s social support found a positive link between adequate social support and mental health outcomes [24–32]. In turn, lacking social support has been associated with negative impacts on people’s well-being [33–36].

Applying the concept of social support to human-animal relationships is a logical extension. When describing the advantages of pet ownership, people will often mention the emotional support and self-esteem gained from the relationship [37]. Pets provide emotional stability and affection during stressful events such as divorce or bereavement [38, 39]. Pets are perceived as always available, predictable in their responses, and non-judgmental [40–43]. In addition, they are considered to be dependent and caring towards their owners with unconditional love [44, 45]. Pets provide tactile comfort [46] and recreational distraction from worries [47, 48]. In contrast with other social interactions, no special social skills are usually required to elicit a positive response from a pet [49]. Pets’ reactions are not based on who the person is or their social competence, which provides a level of ease and sense of relief not usually experienced in other human-human social interactions [44, 50].

Prior evidence suggests that pet dogs in particular may provide social support to humans by contributing to enhanced positive affective states and decreased sadness, anxiety, and loneliness [51–63]. However, not all studies have reported positive effects of dogs on their owners’ well-being [64–66]. In fact, some studies found that dog ownership may increase an owner’s stress levels [44, 67–71]. The mixed results indicated by research to date reveals a gap in knowledge regarding when and how pet dog ownership contributes to greater wellbeing among pet owners.

The context of the COVID-19 pandemic offered a unique opportunity to better understand how dogs may provide social support for their owners and buffer heightened symptoms of stress, anxiety and depression and contribute to happiness. Recently published studies suggest that pet ownership during the pandemic had a positive influence on pet owners. Pet ownership was associated with improved mood [72], reduced loneliness [73, 74], greater social support [75–77], and relieved stress by increased physical exercise [75, 78]. Dog owners reported that their dogs helped them cope with emotional stressors (91.2%) and maintain physical activity (96.4%) during lockdown [78]. Dog walking during confinements may have alleviated stressors and motivated self-care [73]. The results of a non-peer reviewed survey study that explored the relationship, experiences, and concerns of human-pet dog dyads during the pandemic suggested that dogs may help to reduce feelings of distress, anxiety, depression, and isolation [79]. However, recent studies have also reported that pet ownership during the COVID-19 pandemic may have negatively affected people because of limited availability to resources (e.g., veterinary care, pet supplies) [75, 78, 80, 81].

The present study aimed to understand if pet dogs offered their owners social support and contributed to better wellbeing during the COVID-19 pandemic. It was hypothesized that pet dog ownership would act as a buffer against negative impacts caused by the pandemic. Two groups of people (pet dog owners and potential dog owners) were surveyed. Both groups were asked to answer validated psychometric questionnaires on depression, anxiety, and happiness. Other types of pets are also likely to provide social support to humans. However, it is unclear if this support is equivalent and if the psychological mechanisms involved are the same as human-dog relationships. In the context of the COVID-19 pandemic, there is emerging evidence that the relationship and attitude of people towards their pets may vary according to the species [82]. To avoid this potential confound, only dog owners and potential dog owners were included in this survey. Participants also answered questions about how much social support they perceived was available to them, and about their attitude towards and commitment to pets.

## Methods

### Participants

All participants were at least 18 years old, English speaking, had access to a computer, and were living in the United States at the time of the survey. Only one person per household was eligible to take the online survey. People owning other types of pets (e.g., cats, small rodents, rabbits, horses, birds), or who failed to complete the entire survey were excluded. Pet dog owners were defined as people who own at least one dog. Those who owned more than one dog were asked to answer the survey for the one dog that they felt the closest to. Because of the distinct nature of the relationship, we excluded dog owners whose dog was a service, emotional support, or therapy dog. Potential pet dog owners were defined as individuals who did not own a dog at the time of the survey but would be very or extremely interested in owning one in the future. People who did not have dogs and indicated that they were not interested in owning dogs in the future were excluded from the study.

Participants were asked to answer a series of demographic questions about race, ethnicity, employment status, education, annual household income, type of community they live in (urban, suburban, rural), number of dogs they own, age of the dog, and how long they have owned the dog (details can be found in S1 to S16 Tables). Participants in the two groups were then matched by sex, age, place of residence, and by the perceived negative impact that the pandemic had on their finances, emotions, health, and lifestyle.

A third-party research firm (Prodege, El Segundo, CA) recruited participants through multiple strategies including online advertisements via banner ads, referrals from current panelists, and targeted advertising in third-party search engines and websites across the Internet. The study was reviewed by an independent review board, Pearl Pathways (Indianapolis, IN), and received an exemption determination (protocol 20-NEST-101).

The survey was initially administered from November 9^th^ to the 24^th^ of 2020 (753 dog owners and 752 potential dog owners). Three hundred thirty-five (335) dog owners who also owned other pets were inadvertently allowed to complete the survey and were therefore removed from data analysis. In order to rebalance the groups, 335 potential dog owners with similar demographic profiles were also randomly removed from the data set. From February 18^th^ to the 22^nd^ of 2021, an additional 350 dog owners and 350 potential dog owners were surveyed. The final sample is composed of 1,535 participants (768 pet dog owners and 767 potential pet dog owners).

### Psychometric scales

Participants were evaluated using the following six validated psychometric scales. The scales were selected based on their specialized assessments of human-pet relationships, perceived social support, and self-reported psychological states.

#### Pet Attitude Scale [83]

This 18-item scale measures favorableness of attitudes towards pets. Participants answer each item using a 7-point Likert scale (strongly disagree/strongly agree). The total score ranges from 18 (most unfavorable attitude towards pets) to 126 (most favorable attitude towards pets). This scale has high internal consistency and test-retest reliability [83].

#### Miller-Rada Commitment to Pets Scale [84]

This 10-item scale uses a 5-point Likert scale (strongly agree/strongly disagree) to assess how much time, energy, and resources an owner is willing to devote to their pet, even when facing a negative situation (e.g., pet’s destructiveness). The total score varies from 10 (least committed) to 50 points (most committed). The Miller-Rada Commitment to Pets Scale has high internal reliability [84].

#### Multidimensional Scale of Perceived Social Support (MSPSS) [85]

This 12-item, 7-point Likert scale (strongly agree/strongly disagree) instrument measures how much support people perceive they receive from friends, family, and significant others. To calculate a perceived social support score, the responses across all 12 items are summed and the total is divided by 12. There are no established population norms on the MSPSS. However, in this study, we adopted the rule proposed by [86]: scores ranging from 1 to 2.9 are considered low support; scores of 3 to 5 are considered moderate support; and scores greater than 5 are considered high support. The MSPSS is a psychometrically sound instrument with adequate internal and test-retest reliability, strong factorial validity, and moderate construct validity [85].

#### Center for Epidemiologic Studies Depression Scale-Revised (CESD-R) [87]

The 20 items of this 5-point Likert scale are based on the criteria for depression from the Diagnostic and Statistical Manual of Mental Disorders V [88]. Participants are asked how often they have experienced each item in the past two weeks (not at all or less than 1 day/nearly every day for 2 weeks). A score of less than 16 indicates little or no depression and is not considered clinically significant. The cut-off for possible depression is 16. The maximum score is 80. The CESD-R shows strong psychometric properties as demonstrated by its exploratory and confirmatory analyses, internal consistency, and convergent and divergent validity [89].

#### Generalized Anxiety Disorder Scale (GAD-7) [90]

This 7-item, 4-point Likert scale (not at all/nearly every day) measures the severity of generalized anxiety disorder in people. Participants are asked how often they have experienced each item in the past two weeks. Scores of 0 to 9 indicate no to mild anxiety. The cut-off score for anxiety is 10. The maximum score is 21. This scale is an efficient tool to screen for generalized anxiety disorder with high internal consistency, good test-retest reliability and procedural validity, strong diagnostic criterion validity and construct validity [90].

#### Oxford Happiness Questionnaire (OHQ) [91]

This scale provides a broad measure of personal happiness. It contains 29 items and participants answer each item using a 6-point Likert scale (strongly disagree/strongly agree). A personal happiness score, varying from 1 to 6, is obtained by summing the person’s answers and dividing the total by 29. The scores are interpreted as follows (adapted from [92]: less than 2 is “Not happy”; between 2 and 3 is “Somewhat unhappy”; between 3 and 4 is “Not particularly happy/unhappy”; between 4 and 5 is “Rather happy”; greater than 5 is “Very happy”. The OHQ has high internal consistency and good construct validity [93].

### Sample size and power calculations

Based on published data from the CESD-R scale [89], the GAD-7 [94, 95], and the OHQ [96, 97], a sample size of at least 750 participants per group provides sufficient power (actual power 0.80) to detect a 15% difference between the groups (one-tail) at a confidence level of 95%.

### Statistical analysis

Data transformation and descriptive statistics were computed using R [98]. The package openxlsx [99] was used to read and write excel files. The package rstatix [100] was used to perform Mann-Whitney U-tests. The data was composed of two non-normally distributed groups (confirmed with Shapiro test). Therefore, the Mann-Whitney U-test with Bonferroni correction was used (non-parametric alternative to an independent samples t-test) to assess the association between dog ownership and depression, anxiety, and happiness. It was also used to explore the possible links between dog ownership status, pet attachment and commitment, perceived social support, and depression, anxiety, and happiness. Effect size was calculated using Cohen’s d.

## Results

The descriptive statistics of the perceived impact of COVID-19 on finances, emotions, health, and lifestyle of the dog owners and potential dog owners are presented in S17 to S20 Tables. The descriptive statistics of the answers of the dog owners and potential dog owners to the six psychometric scales used in this study are presented in S21 to S26 Tables.

### Perceived negative impact of the pandemic on the participants’ lives

Because the two groups of participants were matched based on their responses to perceived negative impacts of the pandemic, the overall results (dog owners and potential dog owners) are presented in this section. Thirty-three percent of the participants reported that their health had been somewhat to extremely impacted during this period. Forty-five percent indicated that their finances were somewhat to extremely impacted. Sixty-seven percent said that their emotions had been somewhat to extremely impacted. Finally, seventy-two percent reported that their lifestyle had been somewhat to extremely impacted.

### Dog ownership status and depression, anxiety, and happiness

On average, dog owners had a lower depression score (M = 12.41; SD = 14.25), compared to potential dog owners (M = 14.06; SD = 14.86). The difference between the groups was statistically significant (U = 271493.5, p = .018) and the effect size was small (d = 0.07). There was no significant difference (U = 281667, p = .186) between the anxiety scores of the dog owners (M = 4.43; SD = 5.04) and the potential dog owners (M = 4.82; SD = 5.27). There was no significant difference (U = 305278, *p* = .216) between the happiness scores of the dog owners (M = 4.05; SD = 0.89) and the potential dog owners (M = 3.99; SD = 0.91).

### Dog ownership status and attitude towards pets

Dog owners had a significantly more positive attitude towards pets on average compared to the potential dog owner group (U = 358322, p = .000). The average score of the dog owners on the Pet Attitude Scale was 109.93 (SD = 13.49) and the average score of the potential dog owners was 105.94 (SD = 13.16). The effect size of dog ownership on attitude scores was small (d = 0.19). There were no correlations between attitude towards pets and depression, anxiety, and happiness scores (S27 Table).

### Dog ownership status and commitment towards pets

Dog owners, on average, showed significantly greater commitment to pets compared to potential dog owners (U = 355577.5, p = .000). The average score of the dog owners on the Miller-Rada Commitment to Pets Scale was 43.97 (SD = 7.25) and the average score of the potential dog owners was 41.49 (SD = 7.66). The effect size of dog ownership on commitment scores was small (d = 0.18). There were no correlations between commitment towards pets and depression, anxiety, and happiness scores (S28 Table).

### Dog ownership and perceived social support

Dog owners reported having significantly more social support available to them compared to the potential dog owner group (U = 314088.5; p = .042). The average score of the dog owners on the Multidimensional Scale of Perceived Social Support was 5.48 (SD = 1.23) and it was 5.34 (SD = 1.28) for the potential dog owners. The effect size of dog ownership on perceived social support scores was small (d = 0.06).

### Dog ownership, perceived social support, and depression, anxiety, and happiness

We explored *ex post facto* the possible links between dog ownership status, perceived social support, and depression, anxiety, and happiness by dividing dog owners and potential dog owners into groups according to their reported levels of social support: low, moderate, and high. There were no statistical differences in perceived social support and depression, anxiety, and happiness between dog owners and potential dog owners (Table 1). However, the depression score’s mean value of the dog owners was lower than the depression score’s mean value of the potential dog owners for all three levels of social support. The anxiety scores for the dog owners who reported low and moderate levels of perceived social support were lower than the anxiety scores of the potential dog owners for the same levels of perceived social support. Further, the happiness scores of the dog owners were higher than the happiness scores of the potential dog owners for the low and moderate levels.

**Table 1:** Dog owners’ and potential dog owners’ average depression, anxiety, and happiness scores by perceived social support level.

**Depression scores (SD)**				
**Level of social support** [n]	Dog owners [n]	Potential dog owners [n]	Wilcoxon	Z value
Low [77]	29.91 (21.52) [34]	30.93 (19.76) [43]	699	1
Moderate [420]	15.03 (15.60) [198]	18.90 (16.90) [222]	18831.5	.1008
High [1035]	10.33 (12.11) [536]	10.47 (11.44) [502]	129704.5	1
**Anxiety scores (SD)**				
Low	9.53 (7.08)	9.65 (6.60)	719	1
Moderate	5.10 (5.26)	6.59 (6.13)	19050.5	.1584
High	3.86 (4.59)	3.62 (4.18)	135466	1
**Happiness scores (SD)**				
Low	2.86 (0.84)	2.67 (0.86)	831	1
Moderate	3.59 (0.70)	3.55 (0.80)	22427	1
High	4.30 (0.82)	4.30 (0.76)	136765	1

This *ex post facto* exploration suggests that there was a correlation between levels of perceived social support and the depression, anxiety, and happiness scores of the dog owners and potential dog owners. It was not possible to analyze the data statistically because of the large differences between the number of participants in the reported levels of social support (77 in the low group, 420 in the moderate group, and 1032 in the high group). However, it is worth mentioning that the depression scores of the participants with low perceived social support were almost three times higher than the depression scores of the participants with high perceived social support (approximately 30 vs. 10). From a clinical perspective, it is also of significance to note that participants who reported low perceived social support had, on average, depression scores much higher than the cut-off for possible depression of 16 from the CESD-R [87]. Anxiety scores of the participants who reported low perceived social support were about 2.5 times higher than those who reported high perceived social support (approximately 9.6 vs. 3.7). All mean anxiety scores were lower than the cut-off of 10 of the GAD-7 [90]. However, the mean scores of the participants who reported low perceived social support were less than half a point from the cut-off value for possible anxiety. The OHS [91] mean happiness scores of the participants who reported low perceived social support were about 65% lower than those who reported high social support (approximately 2.8 vs. 4.3).

## Discussion

This study adds to the emerging body of literature on human-animal interaction during the COVID-19 pandemic by quantifying the potential benefits of pets on human psychological health and wellbeing. Our results showed that dog owners had significantly lower depression scores than potential dog owners, but the two groups had similar anxiety and happiness scores. In addition, dog owners showed significantly more positive attitude and higher commitment towards pets than potential dog owners. Dog owners also reported a significantly higher degree of perceived social support. Taken together, our results suggest that dog ownership may have provided people with a stronger sense of social support, which in turn may have helped buffer some of the negative psychological impacts caused by the COVID-19 pandemic.

In keeping with previously published research, our results showed the complexity of measuring the association between dog ownership and people’s wellbeing. While dog-ownership or -fostering has been correlated with better depression outcomes [101, 102], other studies have found no correlations [54, 58, 64, 103, 104], or found that pet ownership was associated with higher depression levels [105–108]. In addition, the correlation between pet ownership and depression symptoms was modulated by marital status and sex [109]; unmarried men with pets had the highest depression scores amongst men, while married women with pets had the lowest depression scores amongst women.

Similarly, the correlation between pet ownership and owners’ anxiety symptoms is limited and inconclusive. Some exploratory studies have reported a link between pet ownership and decreased anxiety symptoms in children with autism [110, 111] and in older adults [112]. However, other studies have reported no correlations [113], or increased anxiety in pet owners [106]. Investigating successful aging in older adults, one study reported that dog ownership was positively associated with psychological wellbeing indicators [114]. Still, most research on pet ownership and happiness and related concepts such as quality of life, wellbeing, or life satisfaction found no differences between pet owners and non-pet owners [58, 106, 115–118]. Our study found no link between dog ownership and anxiety and happiness scores. Multiple interfacing lifestyle factors such as physical activity, diet, sleep, stress management, avoidance of substance abuse, and social connection (including pet ownership) modulate depression, anxiety, and happiness. Teasing out their relative importance often proves to be difficult, and this may have been a factor at play in our study.

Our results showed that dog owners reported having more social support available to them compared to potential dog owners. These findings are consistent with other studies documenting that pets provide social support to humans [54, 56–58, 60, 114, 119]. However, when dog owners and potential dog owners were further divided according to their reported levels of social support, there was no difference between the groups in terms of depression, anxiety, and happiness. Also, while dog owners showed a more positive attitude towards and commitment to pets compared to the potential dog owners, the scores were not correlated to depression, anxiety, and happiness. In future research, it may be useful to look further into the quality of the relationship between people and their pets as it may be an important factor to consider. Different pet relationship scales may tap into different aspects of the human-pet relationship. For example, research that utilized Bowlby’s attachment theory [120] and Rogers’ core conditions [121] to examine human-pet relationships found a correlation between secure pet attachment and better quality of life, lower psychological distress, and better psychopathology in pet owners [118]. In a study conducted in the first half of 2020 with cat and dog owners, above average pet attachment was found to be a protective mental health factor for people with moderate to high levels of mental health symptoms. However, high pet attachment in people with severe levels of mental health symptoms was associated with decreased chances of improved mental health [122]. The pet relationship scales used in the current study may not have facilitated insight into the nature of the human-pet relationship that most affects depression, anxiety, and happiness during a crisis such as the COVID-19 pandemic.

The dog owner participants were not asked questions regarding the type of activities they engaged in with their dogs. Doing so may have helped better measure the benefits of owning a dog. In response to the prevalence of contradictory findings in pet ownership benefit studies, a framework was developed to isolate variables within the pet ownership lifestyle by focusing on specific activities associated with pet ownership that could promote clearer results [123]. Other findings suggested that future research could also investigate how specific activities (e.g., dog walking, tactile interaction, meeting pet’s needs) modulate mental health measures like depression, anxiety, and happiness among pet owners [56].

There was a difference between some of our results and what pet owners say when asked about the importance of their pet during the COVID-19 pandemic. For example, in a recent bespoke questionnaire, 58% of respondents reported that their dog helped manage anxiety and 57% reported their dog helped with depression [124]. Seventy-two percent of pet owners indicated that they “would not have been able to get through” the pandemic without their pet [125]. Eighty-seven percent of people indicated that their animal was helping them to cope emotionally during the pandemic; 91% reported that their pets were a significant source of emotional support; and 94% said that their animal had a positive effect on their family [78]. Based on these results, it appears that pets were highly valued and that they positively contributed to the quality of life of their owners in meaningful ways during the pandemic. Why was the dog effect not more evident in the data we collected? One possible explanation is that its effect is real, but its effect size is smaller than the effect of other lifestyle factors that may affect psychological outcomes. Also, when prompted about the role that their dogs may play in specific situations, people may overestimate their actual impact [126]. In all likelihood, people are genuine when they report how much they love their dogs and how much comfort their dogs bring them. However, the dog effect may not be strong enough to completely counterbalance the traumatic impact of major life events such as the loss of a job, a divorce, or, in our case, the COVID-19 pandemic. Similarly, no quantitative correlations were found between pet ownership and loneliness and wellbeing, despite people qualitatively commenting that their pets provided them with social support and a sense of purpose during the pandemic [82].

Pets’ contribution to the wellbeing of people may be more apparent among individuals in precarious states (e.g., high stress, socially isolated) [52]. In our study, 70% of dog owners (and 65% of potential dog owners) reported benefiting from high social support from family and friends and this degree of social support is likely to have provided a buffering effect against the negative impact of the COVID-19 pandemic. Dogs likely contributed positively to the general wellbeing of the dog owner participants, but this effect may have been masked by the social support received by the participants from other people. Therefore, future research focusing on people with low and moderate social support may better capture differences between dog owners and potential dog owners.

An association between levels of perceived social support and average depression, anxiety, and happiness scores among all participants (both dog owners and potential dog owners) was observed in an *ex post facto* analysis that we conducted. Compared to those who reported high perceived social support, people who reported low social support had depression and anxiety scores about twice as high and their happiness scores were notably lower. It is important to point out that this association is solely based on the visual comparison of the mean scores. Yet, this observation is in line with the body of literature on the importance of social support in difficult times [27, 31, 32, 34]. These observations support the suggestion that dog ownership effects might be most measurable within populations of people with low to moderate social support.

The current study was carefully designed to avoid common methodological mistakes in human-animal interaction research [127], but still, some limitations are present. The timing of survey studies is always an important factor to consider. The survey was initially administered in November 2020, a month of anticipation and stress for many Americans headed into the holiday season. It was administered a second time in the spring of 2021. While there were concerns about the effect of the timing differences between the two survey groups, carefully controlled recruitment indicates that there were no demographic differences between the groups. Of note, the US government only issued voluntary public health recommendations aimed at controlling the transmission of the virus. There were no federally unified or enforced responses. What the implemented mandates were and when they were implemented varied by states and by localities [128]. It is possible to imagine that people who lived in states or localities where the restrictions were more extensive or lasted longer would have benefited differently from the presence of their dogs compared to people who lived in places where the restrictions were fewer or shorter in duration. Because of this great variability, we were not able to analyze potential differences between areas.

A large number of participants were used, and potential confounders were controlled for (e.g., sex, age, place of residence, perceived negative impact that the pandemic had on finances, emotions, health, and lifestyle). Participants in both groups were also balanced on all demographic variables. The limitation of participants’ self-reported information is acknowledged, however validated psychometric tools that have been widely and successfully used by many researchers were used. To avoid selection bias, participants were recruited by an independent third-party.

It was not possible to use a true experimental design where half the participants would be provided with a dog and half would not get a dog. Usually, research investigating the possible benefits of dog ownership compare a group of dog owners to a group of non-dog owners. Instead, a quasi-experimental design was developed such that dog owners were compared to a specific group of non-dog owners, i.e., people who did not own a dog at the time of the survey but who indicated that they were very interested in acquiring one. This selection criteria may offer a more effective comparison than including a group of participants who are not interested in dogs and who made the decision not to live with one. It is fair to assume that these people would gain no benefit from owning pet dogs and that they seek and receive social support through other means. In future research, we propose that in addition to comparing dog owners to potential dog owners, consideration should be given to including participants with more diverse pet attachment and commitment scores. In this sample population, most participants scored high on both measures and this may not be representative of the general population.

## Conclusion

The COVID-19 pandemic has negatively affected diverse populations [129–134] and our results provide evidence that pet owners and potential pet owners have also been impacted. Our results show that pet dog owners were significantly less depressed than non-pet owners during the COVID-19 pandemic. They are attached and committed to their dogs and they reported more social support available to them. Our work adds to the corpus of scientific literature demonstrating that pet dogs may positively contribute to the wellbeing of owners during difficult times. However, more work is needed to better understand the relationship between pet ownership and social support as modulators of owner wellbeing. Future research should focus on people with low and moderate social support and include owners with diverse dog attachment levels.

## Supporting information

S1 Table Age of participants. (DOCX)

S2 Table Sex/gender of participants. (DOCX)

S3 Table US state of residence. (DOCX)

S4 Table US division of residence. (DOCX)

S5 Table US region of residence. (DOCX)

S6 Table Number of dogs. (DOCX)

S7 Table Ownership duration. (DOCX)

S8 Table Age of dogs. (DOCX)

S9 Table Number of people in household. (DOCX)

S10 Table Marital status. (DOCX)

S11 Table Education level. (DOCX)

S12 Table Employment status. (DOCX)

S13 Table Annual income. (DOCX)

S14 Table Metropolitan-nonmetropolitan classification. (DOCX)

S15 Table Racial or ethnic background. (DOCX)

S16 Table Hispanic origin or descent. (DOCX)

S17 Table Perceived impact of Covid-19 on finances. (DOCX)

S18 Table Perceived impact of Covid-19 on emotions. (DOCX)

S19 Table Perceived impact of Covid-19 on health. (DOCX)

S20 Table Perceived impact of Covid-19 on lifestyle. (DOCX)

S21 Table Pet Attitude Scale descriptive statistics. (DOCX)

S22 Table Miller-Rada Commitment to Pets Scale descriptive statistics. (DOCX)

S23 Table Multidimensional Scale of Perceived Social Support descriptive statistics. (DOCX)

S24 Table Center for Epidemiologic Studies Depression Scale-Revised descriptive statistics. (DOCX)

S25 Table Generalized Anxiety Disorder Scale descriptive statistics. (DOCX)

S26 Table Oxford Happiness Scale descriptive statistics. (DOCX)

S27 Table Correlations between attitude towards pets and depression, anxiety, and happiness scores. (DOCX)

S28 Table Correlations between commitment to pets and depression, anxiety, and happiness scores. (DOCX)

S1 Data (XLSX)
