# Supplementary material for: Depression, anxiety, and happiness in dog owners and potential dog owners during the COVID-19 pandemic in the United States
Source: PLoS One. 2021 Dec 15;16(12):e0260676. doi: 10.1371/journal.pone.0260676 (PMC8673598; doi:10.1371/journal.pone.0260676)
Supplement: S1 Table — (DOCX) [file pone.0260676.s001.docx]

**S1 Table. Age of participants.**

The average age of the dog owners was 45.43 (min=18; max=82) and the average age of the potential dog owners was 42.41 (min=18; max=99). Additional information about the age distribution of the participants is presented in the table.

| Age Group | Dog owners | | | | | | Potential dog owners | | | | | |
| --- | --- | --- | --- | --- | --- | --- | --- | --- | --- | --- | --- | --- |
|  | 11/2020 | | 02/2021 | | Final sample | | 11/2020 | | 02/2021 | | Final sample | |
|  | n | % | n | % | n | % | n | % | n | % | n | % |
| 18-34 | 125 | 29.90 | 95 | 27.14 | 220 | 28.65 | 126 | 30.22 | 95 | 27.14 | 221 | 28.81 |
| 35-44 | 88 | 21.05 | 81 | 23.14 | 169 | 22.00 | 88 | 21.10 | 80 | 22.86 | 168 | 21.90 |
| 45-54 | 71 | 16.99 | 58 | 16.57 | 129 | 16.80 | 71 | 17.03 | 58 | 16.57 | 129 | 16.82 |
| 55-64 | 87 | 20.81 | 77 | 22.00 | 164 | 21.35 | 85 | 20.38 | 77 | 22.00 | 162 | 21.12 |
| 65 + | 47 | 11.24 | 39 | 11.14 | 86 | 11.20 | 47 | 11.27 | 40 | 11.43 | 87 | 11.34 |
| Total | 418 | 99.9* | 350 | 99.99* | 768 | 100 | 417 | 100 | 350 | 100 | 767 | 99.99* |

* Total not equal to 100% due to rounding error.
