# Supplementary material for: Depression, anxiety, and happiness in dog owners and potential dog owners during the COVID-19 pandemic in the United States
Source: PLoS One. 2021 Dec 15;16(12):e0260676. doi: 10.1371/journal.pone.0260676 (PMC8673598; doi:10.1371/journal.pone.0260676)
Supplement: S2 Table — (DOCX) [file pone.0260676.s002.docx]

**S2 Table. Sex/gender of participants.**

The sex/gender distribution in the dog owner and the potential dog owner groups were almost identical. Both groups had 447 female respondents; 320 males were in the dog owner group and 319 males were in the potential dog owner group; one person preferred not to answer the question in the dog owner group and one person identified as other in the potential dog owner group.

| Sex/  gender | Dog owners | | | | | | Potential dog owners | | | | | |
| --- | --- | --- | --- | --- | --- | --- | --- | --- | --- | --- | --- | --- |
|  | 11/2020 | | 02/2021 | | Final sample | | 11/2020 | | 02/2021 | | Final sample | |
|  | n | % | n | % | n | % | n | % | n | % | n | % |
| Female | 241 | 57.66 | 206 | 58.86 | 447 | 58.20 | 241 | 57.79 | 206 | 58.83 | 447 | 58.28 |
| Male | 176 | 42.11 | 144 | 41.14 | 320 | 41.67 | 176 | 42.21 | 143 | 40.86 | 319 | 41.59 |
| Other | 0 | 0.00 | 0 | 0.00 | 0 | 0.00 | 0 | 0.00 | 1 | 0.29 | 1 | 0.13 |
| Prefer not to answer | 1 | 0.24 | 0 |  | 1 | 0.13 | 0 | 0.00 | 0 | 0.00 | 0 | 0.00 |
| Total | 418 | 100.01* | 350 | 100 | 768 | 100 | 417 | 100 | 350 | 99.98* | 767 | 100 |

* Total not equal to 100% due to rounding error.
