# Supplementary material for: Depression, anxiety, and happiness in dog owners and potential dog owners during the COVID-19 pandemic in the United States
Source: PLoS One. 2021 Dec 15;16(12):e0260676. doi: 10.1371/journal.pone.0260676 (PMC8673598; doi:10.1371/journal.pone.0260676)
Supplement: S3 Table — (DOCX) [file pone.0260676.s003.docx]

**S3 Table. US State of residence.**

|  | Dog owners | | | Potential dog owners | | |
| --- | --- | --- | --- | --- | --- | --- |
|  | 11/2020 | 02/2021 | Final sample | 11/2020 | 02/2021 | Final sample |
|  | n | n | n | n | n | n |
| Alabama | 2 | 3 | 5 | 6 | 7 | 13 |
| Alaska | 0 | 2 | 2 | 2 | 0 | 2 |
| Arizona | 8 | 6 | 14 | 12 | 5 | 17 |
| Arkansas | 4 | 5 | 9 | 5 | 1 | 6 |
| California | 40 | 30 | 70 | 42 | 27 | 69 |
| Colorado | 11 | 9 | 20 | 5 | 1 | 6 |
| Connecticut | 6 | 6 | 12 | 4 | 3 | 7 |
| Delaware | 4 | 1 | 5 | 5 | 2 | 7 |
| Florida | 28 | 24 | 52 | 24 | 25 | 49 |
| Georgia | 17 | 9 | 26 | 10 | 14 | 24 |
| Hawaii | 3 | 1 | 4 | 3 | 1 | 4 |
| Idaho | 2 | 2 | 4 | 2 | 4 | 6 |
| Illinois | 23 | 21 | 44 | 24 | 21 | 45 |
| Indiana | 3 | 6 | 9 | 9 | 10 | 19 |
| Iowa | 8 | 5 | 13 | 4 | 6 | 10 |
| Kansas | 8 | 4 | 12 | 1 | 0 | 1 |
| Kentucky | 9 | 8 | 17 | 10 | 6 | 16 |
| Louisiana | 8 | 5 | 13 | 5 | 5 | 10 |
| Maine | 2 | 0 | 2 | 2 | 0 | 2 |
| Maryland | 6 | 9 | 15 | 9 | 10 | 19 |
| Massachusetts | 9 | 7 | 16 | 12 | 14 | 26 |
| Michigan | 12 | 10 | 22 | 15 | 13 | 28 |
| Minnesota | 6 | 7 | 13 | 5 | 5 | 10 |
| Mississippi | 1 | 4 | 5 | 5 | 1 | 6 |
| Missouri | 7 | 8 | 15 | 8 | 8 | 16 |
| Montana | 1 | 1 | 2 | 1 | 0 | 1 |
| Nebraska | 1 | 3 | 4 | 3 | 1 | 4 |
| Nevada | 5 | 3 | 8 | 4 | 4 | 8 |
| New Hampshire | 1 | 1 | 2 | 1 | 3 | 4 |
| New Jersey | 14 | 12 | 26 | 10 | 15 | 25 |
| New Mexico | 2 | 0 | 2 | 2 | 1 | 3 |
| New York | 24 | 17 | 41 | 29 | 16 | 45 |
| North Carolina | 10 | 13 | 23 | 15 | 8 | 23 |
| North Dakota | 1 | 0 | 1 | 1 | 3 | 4 |
| Ohio | 13 | 11 | 24 | 14 | 14 | 28 |
| Oklahoma | 2 | 3 | 5 | 5 | 5 | 10 |
| Oregon | 5 | 1 | 6 | 5 | 8 | 13 |
| Pennsylvania | 20 | 20 | 40 | 17 | 15 | 32 |
| Rhode Island | 1 | 3 | 4 | 2 | 1 | 3 |
| South Carolina | 8 | 4 | 12 | 6 | 12 | 18 |
| South Dakota | 0 | 4 | 4 | 1 | 3 | 4 |
| Tennessee | 12 | 9 | 21 | 7 | 5 | 12 |
| Texas | 26 | 26 | 52 | 28 | 15 | 43 |
| Utah | 5 | 1 | 6 | 4 | 3 | 7 |
| Vermont | 1 | 0 | 1 | 0 | 0 | 0 |
| Virginia | 12 | 7 | 19 | 12 | 15 | 27 |
| Washington | 6 | 6 | 12 | 5 | 10 | 15 |
| West Virginia | 6 | 2 | 8 | 3 | 0 | 3 |
| Wisconsin | 14 | 11 | 25 | 12 | 4 | 16 |
| Wyoming | 1 | 0 | 1 | 1 | 0 | 1 |
| Total | 418 | 350 | 768 | 417 | 350 | 767 |
