# Supplementary material for: Depression, anxiety, and happiness in dog owners and potential dog owners during the COVID-19 pandemic in the United States
Source: PLoS One. 2021 Dec 15;16(12):e0260676. doi: 10.1371/journal.pone.0260676 (PMC8673598; doi:10.1371/journal.pone.0260676)
Supplement: S4 Table — (DOCX) [file pone.0260676.s004.docx]

**S4 Table. US division of residence.**

|  | Dog owners | | | | | | Potential dog owners | | | | | |
| --- | --- | --- | --- | --- | --- | --- | --- | --- | --- | --- | --- | --- |
|  | 11/2020 | | 02/2021 | | Final sample | | 11/2020 | | 02/2021 | | Final sample | |
|  | n | % | n | % | n | % | n | % | n | % | n | % |
| New England | 20 | 4.78 | 17 | 4.86 | 37 | 4.82 | 21 | 5.04 | 21 | 6.00 | 42 | 5.48 |
| Middle Atlantic | 58 | 13.88 | 49 | 14.00 | 107 | 13.93 | 56 | 13.43 | 46 | 13.14 | 102 | 13.30 |
| East North Central | 65 | 15.55 | 59 | 16.86 | 124 | 16.15 | 74 | 17.75 | 62 | 17.71 | 136 | 17.73 |
| West North Central | 31 | 7.42 | 31 | 8.86 | 62 | 8.07 | 23 | 5.52 | 26 | 7.43 | 49 | 6.39 |
| South Atlantic | 91 | 21.77 | 69 | 19.71 | 160 | 20.83 | 84 | 20.14 | 86 | 24.57 | 170 | 22.16 |
| East South Central | 24 | 5.74 | 24 | 6.86 | 48 | 6.25 | 28 | 6.71 | 19 | 5.43 | 47 | 6.13 |
| West South Central | 40 | 9.57 | 39 | 11.14 | 79 | 10.29 | 43 | 10.31 | 26 | 7.43 | 69 | 9.00 |
| Mountain | 35 | 8.37 | 22 | 6.29 | 57 | 7.42 | 31 | 7.43 | 18 | 5.14 | 49 | 6.39 |
| Pacific | 54 | 12.92 | 40 | 11.43 | 94 | 12.24 | 57 | 13.67 | 46 | 13.14 | 103 | 13.43 |
| Total | 418 | 100 | 350 | 100.01* | 768 | 100 | 417 | 100 | 350 | 99.99* | 767 | 100.01* |

* Total not equal to 100% due to rounding error.
