# Supplementary material for: Depression, anxiety, and happiness in dog owners and potential dog owners during the COVID-19 pandemic in the United States
Source: PLoS One. 2021 Dec 15;16(12):e0260676. doi: 10.1371/journal.pone.0260676 (PMC8673598; doi:10.1371/journal.pone.0260676)
Supplement: S5 Table — (DOCX) [file pone.0260676.s005.docx]

**S5 Table. US region of residence.**

|  | Dog owners | | | | | | Potential dog owners | | | | | |
| --- | --- | --- | --- | --- | --- | --- | --- | --- | --- | --- | --- | --- |
|  | 11/2020 | | 02/2021 | | Final sample | | 11/2020 | | 02/2021 | | Final sample | |
|  | n | % | n | % | n | % | n | % | n | % | n | % |
| Midwest | 96 | 22.97 | 90 | 25.71 | 186 | 24.22 | 97 | 23.26 | 88 | 25.14 | 185 | 24.12 |
| Northeast | 78 | 18.66 | 66 | 18.86 | 144 | 18.75 | 77 | 18.47 | 67 | 19.14 | 144 | 18.77 |
| South | 155 | 37.08 | 132 | 37.71 | 287 | 37.37 | 155 | 37.17 | 131 | 37.43 | 286 | 37.29 |
| West** | 89 | 21.29 | 62 | 17.71 | 151 | 19.66 | 88 | 21.10 | 64 | 18.29 | 152 | 19.82 |
| Total | 418 | 100 | 350 | 99.99* | 768 | 100 | 417 | 100 | 350 | 100 | 767 | 100 |

* Total not equal to 100% due to rounding error.

** Includes Alaska and Hawaii
