# Supplementary material for: Depression, anxiety, and happiness in dog owners and potential dog owners during the COVID-19 pandemic in the United States
Source: PLoS One. 2021 Dec 15;16(12):e0260676. doi: 10.1371/journal.pone.0260676 (PMC8673598; doi:10.1371/journal.pone.0260676)
Supplement: S6 Table — (DOCX) [file pone.0260676.s006.docx]

**S6 Table. Number of dogs.**

Seventy-four percent (74.35%) of dog owners possessed one dog. Twenty percent (20.31%) owned two dogs. The rest of the dog owners (5.34%) owned three or more dogs. Potential dog owners did not own dogs. Participants of both groups did not own any other pets as per the study’s exclusion criteria.

|  | November 2020 | | February 2021 | | Final sample | |
| --- | --- | --- | --- | --- | --- | --- |
|  | n | % | n | % | n | % |
| 1 | 312 | 74.64 | 259 | 74.00 | 571 | 74.35 |
| 2 | 86 | 20.57 | 70 | 20.00 | 156 | 20.31 |
| 3 | 17 | 4.07 | 14 | 4.00 | 31 | 4.04 |
| 4 | 3 | 0.72 | 5 | 1.43 | 8 | 1.04 |
| 5+ | 0 | 0.00 | 2 | 0.57 | 2 | 0.26 |
| Total | 418 | 100 | 350 | 100 | 768 | 100 |
