# Supplementary material for: Depression, anxiety, and happiness in dog owners and potential dog owners during the COVID-19 pandemic in the United States
Source: PLoS One. 2021 Dec 15;16(12):e0260676. doi: 10.1371/journal.pone.0260676 (PMC8673598; doi:10.1371/journal.pone.0260676)
Supplement: S7 Table — (DOCX) [file pone.0260676.s007.docx]

**S7 Table. Ownership duration.**

Eleven percent (11.2%) of dog owners had their dog for 12 months or less. Twenty-five percent (24.61%) owned their dog for 1 to 3 years. Twenty-eight percent (28.13%) owned their dog for 4 to 6 years. The remaining dog owners (36.07%) had their dog for 7 or more years.

|  | November 2020 | | February 2021 | | Final sample | |
| --- | --- | --- | --- | --- | --- | --- |
|  | n | % | n | % | n | % |
| 12 month or less | 46 | 11.00 | 40 | 11.43% | 86 | 11.20 |
| 1-3 years | 103 | 24.64 | 86 | 24.57 | 189 | 24.61 |
| 4-6 years | 119 | 28.47 | 97 | 27.71 | 216 | 28.13 |
| 7-9 years | 68 | 16.27 | 56 | 16.00 | 124 | 16.15 |
| 10-12 years | 57 | 13.64 | 52 | 14.86 | 109 | 14.19 |
| 13 years + | 25 | 5.98 | 19 | 5.43 | 44 | 5.73 |
| Total | 418 | 100.00 | 350 | 100.00 | 768 | 100.01* |
