# Supplementary material for: Depression, anxiety, and happiness in dog owners and potential dog owners during the COVID-19 pandemic in the United States
Source: PLoS One. 2021 Dec 15;16(12):e0260676. doi: 10.1371/journal.pone.0260676 (PMC8673598; doi:10.1371/journal.pone.0260676)
Supplement: S8 Table — (DOCX) [file pone.0260676.s008.docx]

**S8 Table. Age of dogs.**

This Table presents the age distribution of the dogs owned by the participants.

|  | November 2020 | | February 2021 | | Final sample | |
| --- | --- | --- | --- | --- | --- | --- |
|  | n | % | n | % | n | % |
| 12 month or less | 27 | 6.46 | 22 | 6.29 | 49 | 6.38 |
| 13 months-3 years | 92 | 22.01 | 80 | 22.86 | 172 | 22.40 |
| 4-6 years | 128 | 30.62 | 100 | 28.57 | 228 | 29.69 |
| 7-9 years | 65 | 15.55 | 62 | 17.71 | 127 | 16.54 |
| 10-12 years | 72 | 17.22 | 58 | 16.57 | 130 | 16.93 |
| 13 years + | 34 | 8.13 | 28 | 8.00 | 62 | 8.07 |
| Total | 418 | 99.99* | 350 | 100 | 768 | 100.01* |

* Total not equal to 100% due to rounding error.
