# Supplementary material for: Depression, anxiety, and happiness in dog owners and potential dog owners during the COVID-19 pandemic in the United States
Source: PLoS One. 2021 Dec 15;16(12):e0260676. doi: 10.1371/journal.pone.0260676 (PMC8673598; doi:10.1371/journal.pone.0260676)
Supplement: S9 Table — (DOCX) [file pone.0260676.s009.docx]

**S9 Table. Number of people in household.**

Sixteen percent (16.15%) of dog owners and twenty-seven percent (26.73%) of potential dog owners lived by themselves. Forty percent (39.58%) of dog owners and thirty-five percent (34.81%) of potential dog owners lived with one person. Forty-four percent (44.26%) of dog owners and thirty-eight percent (38.47%) of potential dog owners lived with two or more people.

|  | Dog owners | | | | | | Potential dog owners | | | | | |
| --- | --- | --- | --- | --- | --- | --- | --- | --- | --- | --- | --- | --- |
|  | 11/2020 | | 02/2021 | | Final sample | | 11/2020 | | 02/2021 | | Final sample | |
|  | n | % | n | % | n | % | n | % | n | % | n | % |
| 1 | 68 | 16.27 | 56 | 16.00 | 124 | 16.15 | 122 | 29.26 | 83 | 23.71 | 205 | 26.73 |
| 2 | 164 | 39.23 | 140 | 40.00 | 304 | 39.58 | 142 | 34.05 | 125 | 35.71 | 267 | 34.81 |
| 3 | 81 | 19.38 | 79 | 22.57 | 160 | 20.83 | 72 | 17.27 | 53 | 15.14 | 125 | 16.30 |
| 4 | 72 | 17.23 | 45 | 12.86 | 117 | 15.23 | 45 | 10.79 | 58 | 16.57 | 103 | 13.43 |
| 5 + | 33 | 7.90 | 30 | 8.57 | 63 | 8.20 | 36 | 8.63 | 31 | 8.86 | 67 | 8.74 |
| Total | 418 | 100.01* | 350 | 100 | 768 | 99.99* | 417 | 100 | 350 | 99.99* | 767 | 100.01* |
