# Supplementary material for: Depression, anxiety, and happiness in dog owners and potential dog owners during the COVID-19 pandemic in the United States
Source: PLoS One. 2021 Dec 15;16(12):e0260676. doi: 10.1371/journal.pone.0260676 (PMC8673598; doi:10.1371/journal.pone.0260676)
Supplement: S10 Table — (DOCX) [file pone.0260676.s010.docx]

**S10 Table. Marital status.**

Fifty-six percent (56.12%) of dog owners and forty-five percent (44.59%) of potential dog owners were married. Twenty-four percent (24.48%) of dog owners and thirty-three percent (33.38%) of potential dog owners were single. The remaining dog owners (19.40%) and potential dog owners (22.03%) were divorced, in a domestic partnership, separated, widowed, or preferred not to answer the question.

|  | Dog owners | | | | | | Potential dog owners | | | | | |
| --- | --- | --- | --- | --- | --- | --- | --- | --- | --- | --- | --- | --- |
|  | 11/2020 | | 02/2021 | | Final sample | | 11/2020 | | 02/2021 | | Final sample | |
|  | n | % | n | % | n | % | n | % | n | % | n | % |
| Divorced | 28 | 6.70 | 27 | 7.71 | 55 | 7.16 | 45 | 10.79 | 31 | 8.86 | 76 | 9.91 |
| Domestic partnership | 28 | 6.70 | 28 | 8.00 | 56 | 7.29 | 38 | 9.11 | 20 | 5.71 | 58 | 7.56 |
| Married | 233 | 55.74 | 198 | 56.57 | 431 | 56.12 | 170 | 40.77 | 172 | 49.14 | 342 | 44.59 |
| Separated | 9 | 2.15 | 4 | 1.14 | 13 | 1.69 | 3 | 0.72 | 7 | 2.0 | 10 | 1.30 |
| Single/never married | 106 | 25.36 | 82 | 23.43 | 188 | 24.48 | 151 | 36.21 | 105 | 30.00 | 256 | 33.38 |
| Widowed | 13 | 3.11 | 11 | 3.14 | 24 | 3.13 | 8 | 1.92 | 13 | 3.71 | 21 | 2.74 |
| No answer | 1 | 0.24 | 0 | 0.00 | 1 | 0.13 | 2 | 0.48 | 2 | 0.57 | 4 | 0.52 |
| Total | 418 | 100 | 350 | 99.99* | 768 | 100 | 417 | 100 | 350 | 99.99* | 767 | 100 |

* Total not equal to 100% due to rounding error.
