# Supplementary material for: Depression, anxiety, and happiness in dog owners and potential dog owners during the COVID-19 pandemic in the United States
Source: PLoS One. 2021 Dec 15;16(12):e0260676. doi: 10.1371/journal.pone.0260676 (PMC8673598; doi:10.1371/journal.pone.0260676)
Supplement: S11 Table — (DOCX) [file pone.0260676.s011.docx]

**S11 Table. Education level.**

Thirty-six percent (35.94%) of dog owners and thirty-two percent (31.55%) of potential dog owners graduated from college. Thirty percent (29.69%) of dog owners and twenty-eight percent (27.77%) of potential dog owners reported having some college or an associate degree. Eighteen percent (18.23%) of dog owners and twenty-five percent (25.29%) of potential dog owners had completed post-graduate work or degree. The remaining dog owners (16.14%) and potential dog owners (15.39%) had some high school or less, graduated from high school or equivalent, or did not answer the question.

|  | Dog owners | | | | | | Potential dog owners | | | | | |
| --- | --- | --- | --- | --- | --- | --- | --- | --- | --- | --- | --- | --- |
|  | 11/2020 | | 02/2021 | | Final sample | | 11/2020 | | 02/2021 | | Final sample | |
|  | n | % | n | % | n | % | n | % | n | % | n | % |
| Some high school or less | 3 | 0.72 | 8 | 2.29 | 11 | 1.43 | 4 | 0.96 | 8 | 2.29 | 12 | 1.57 |
| Graduated from high school or equivalent (GED) | 68 | 16.27 | 44 | 12.57 | 112 | 14.58 | 63 | 15.11 | 42 | 12.00 | 105 | 13.69 |
| Some college/associate degree | 121 | 28.95 | 107 | 30.57 | 228 | 29.69 | 120 | 28.78 | 93 | 26.57 | 213 | 27.77 |
| Graduated college | 151 | 36.12 | 125 | 35.71 | 276 | 35.94 | 126 | 30.22 | 116 | 33.14 | 242 | 31.55 |
| Post-graduate work or degree | 74 | 17.70 | 66 | 18.86 | 140 | 18.23 | 103 | 24.70 | 91 | 26.00 | 194 | 25.29 |
| No answer | 1 | 0.24 | 0 | 0.00 | 1 | 0.13 | 1 | 0.24 | 0 | 0.00 | 1 | 0.13 |
| Total | 418 | 100 | 350 | 100 | 768 | 100 | 417 | 100.01* | 350 | 100 | 767 | 100 |

* Total not equal to 100% due to rounding error.
