# Supplementary material for: Depression, anxiety, and happiness in dog owners and potential dog owners during the COVID-19 pandemic in the United States
Source: PLoS One. 2021 Dec 15;16(12):e0260676. doi: 10.1371/journal.pone.0260676 (PMC8673598; doi:10.1371/journal.pone.0260676)
Supplement: S12 Table — (DOCX) [file pone.0260676.s012.docx]

**S12 Table. Employment status.**

At the time of the survey, fifty percent (49.74%) of the dog owners and forty-four percent (44.33%) of potential dog owners were working full time. Sixteen percent (16.28%) of dog owners and twenty-one percent (20.99%) of potential dog owners were unemployed. Seventeen percent (17.45%) of dog owners and eighteen percent (18.38%) of potential dog owners were retired. The remaining dog owners (16.54%) and potential dog owners (16.29%) were working part time, were students, or did not answer the question.

|  | Dog owners | | | | | | Potential dog owners | | | | | |
| --- | --- | --- | --- | --- | --- | --- | --- | --- | --- | --- | --- | --- |
|  | 11/2020 | | 02/2021 | | Final sample | | 11/2020 | | 02/2021 | | Final sample | |
|  | n | % | n | % | n | % | n | % | n | % | n | % |
| Full time | 204 | 48.80 | 178 | 50.86 | 382 | 49.74 | 177 | 42.45 | 163 | 46.57 | 340 | 44.33 |
| Part time | 41 | 9.81 | 44 | 12.57 | 85 | 11.07 | 44 | 10.55 | 36 | 10.29 | 80 | 10.43 |
| Retired | 71 | 16.99 | 63 | 18.00 | 134 | 17.45 | 69 | 16.55 | 72 | 20.57 | 141 | 18.38 |
| Student | 19 | 4.55 | 11 | 3.14 | 30 | 3.91 | 25 | 6.00 | 13 | 3.71 | 38 | 4.95 |
| Unemployed | 76 | 18.18 | 49 | 14.00 | 125 | 16.28 | 96 | 23.02 | 65 | 18.57 | 161 | 20.99 |
| No answer | 7 | 1.67 | 5 | 1.43 | 12 | 1.56 | 6 | 1.44 | 1 | 0.30 | 7 | 0.91 |
| Total | 418 | 100 | 350 | 100 | 768 | 100.01* | 417 | 100.01* | 350 | 100.01* | 767 | 99.99* |

* Total not equal to 100% due to rounding error.
