# Supplementary material for: Depression, anxiety, and happiness in dog owners and potential dog owners during the COVID-19 pandemic in the United States
Source: PLoS One. 2021 Dec 15;16(12):e0260676. doi: 10.1371/journal.pone.0260676 (PMC8673598; doi:10.1371/journal.pone.0260676)
Supplement: S13 Table — (DOCX) [file pone.0260676.s013.docx]

**S13 Table. Annual income.**

Twenty-two percent (22.40%) of dog owners and twenty-one percent (20.73%) of potential dog owners reported an annual household income of between $50,000 to $74,999. Nineteen percent of dog owners (19.27%) and potential dog owners (19.30%) reported an annual household income of $75,000 to $99,999. Fourteen percent (13.93%) of dog owners and twenty percent (19.56%) of potential dog owners reported an annual household income of $30,000 to $49,999.

|  | Dog owners | | | | | | Potential dog owners | | | | | |
| --- | --- | --- | --- | --- | --- | --- | --- | --- | --- | --- | --- | --- |
|  | 11/2020 | | 02/2021 | | Final sample | | 11/2020 | | 02/2021 | | Final sample | |
|  | n | % | n | % | n | % | n | % | n | % | n | % |
| Less than $30,000 | 55 | 13.16 | 39 | 11.14 | 94 | 12.24 | 54 | 12.95 | 60 | 17.14 | 114 | 14.86 |
| $30,000-$49,999 | 66 | 15.79 | 41 | 11.71 | 107 | 13.93 | 93 | 22.30 | 57 | 16.29 | 150 | 19.56 |
| $50,000-$74,999 | 90 | 21.53 | 82 | 23.43 | 172 | 22.40 | 98 | 23.50 | 61 | 17.43 | 159 | 20.73 |
| $75,000-$99,999 | 67 | 16.03 | 81 | 23.14 | 148 | 19.27 | 81 | 19.42 | 67 | 19.14 | 148 | 19.30 |
| $100,000-$124,999 | 45 | 10.77 | 44 | 12.57 | 89 | 11.59 | 26 | 6.24 | 35 | 10.00 | 61 | 7.95 |
| $125,000-$149,999 | 32 | 7.66 | 23 | 6.57 | 55 | 7.16 | 20 | 4.80 | 25 | 7.14 | 45 | 5.87 |
| $150,000-$199,999 | 26 | 6.22 | 16 | 4.57 | 42 | 5.47 | 23 | 5.52 | 22 | 6.29 | 45 | 5.87 |
| $200,000 or more | 14 | 3.35 | 17 | 4.86 | 31 | 4.04 | 10 | 2.40 | 13 | 3.71 | 23 | 3.00 |
| No answer | 23 | 5.50 | 7 | 2.00 | 30 | 3.91 | 12 | 2.88 | 10 | 2.86 | 22 | 2.87 |
| Total | 418 | 100.01* | 350 | 99.99* | 768 | 100.01* | 417 | 100.01* | 350 | 100 | 767 | 100.01 |
