# Supplementary material for: Depression, anxiety, and happiness in dog owners and potential dog owners during the COVID-19 pandemic in the United States
Source: PLoS One. 2021 Dec 15;16(12):e0260676. doi: 10.1371/journal.pone.0260676 (PMC8673598; doi:10.1371/journal.pone.0260676)
Supplement: S14 Table — (DOCX) [file pone.0260676.s014.docx]

**S14 Table. Metropolitan-nonmetropolitan classification.**

When asked to describe where they lived, twenty-one percent (20.70%) of dog owners and sixteen percent (16.17%) of potential dog owners reported living in a rural community. Fifty-eight percent (57.94%) of dog owners and fifty-six percent (55.93%) of potential dog owners lived in a suburban community. Twenty-one percent (21.35%) of dog owners and twenty-eight percent (27.90%) of potential dog owners lived in an urban community (S1 Table 12).

|  | Dog owners | | | | | | Potential dog owners | | | | | |
| --- | --- | --- | --- | --- | --- | --- | --- | --- | --- | --- | --- | --- |
|  | 11/2020 | | 02/2021 | | Final sample | | 11/2020 | | 02/2021 | | Final sample | |
|  | n | % | n | % | n | % | n | % | n | % | n | % |
| Rural | 83 | 19.86 | 76 | 21.71 | 159 | 20.70 | 72 | 17.27 | 52 | 14.86 | 124 | 16.17 |
| Suburban | 250 | 59.81 | 195 | 55.71 | 445 | 57.94 | 230 | 55.16 | 199 | 56.86 | 429 | 55.93 |
| Urban | 85 | 20.33 | 79 | 22.57 | 164 | 21.35 | 115 | 27.58 | 99 | 28.29 | 214 | 27.90 |
| Total | 418 | 100 | 350 | 99.99* | 768 | 99.99* | 417 | 100.01* | 350 | 100.01 | 767 | 100 |

* Total not equal to 100% due to rounding error.
