# Supplementary material for: Depression, anxiety, and happiness in dog owners and potential dog owners during the COVID-19 pandemic in the United States
Source: PLoS One. 2021 Dec 15;16(12):e0260676. doi: 10.1371/journal.pone.0260676 (PMC8673598; doi:10.1371/journal.pone.0260676)
Supplement: S15 Table — (DOCX) [file pone.0260676.s015.docx]

**S15 Table. Racial or ethnic background.**

When asked to describe their racial or ethnic background, eighty-five percent (84.77%) of dog owners and seventy-eight percent (77.84%) of potential dog owners reported being White or Caucasian. Seven percent (7.29%) of dog owners and ten percent (10.43%) of potential dog owners identified as Asian or Pacific Islander. Four percent (4.30%) of dog owners and eight percent (7.56%) of potential dog owners reported being Black or African American. One percent of dog owners (0.78%) and potential dog owners (0.91%) identified as Native American or American Indian. The remaining participants (dog owners=2.86%; potential dog owners=3.26%) preferred not to say or answered other.

|  | Dog owners | | | | | | Potential dog owners | | | | | |
| --- | --- | --- | --- | --- | --- | --- | --- | --- | --- | --- | --- | --- |
|  | 11/2020 | | 02/2021 | | Final sample | | 11/2020 | | 02/2021 | | Final sample | |
|  | n | % | n | % | n | % | n | % | n | % | n | % |
| Asian/Pacific Islander | 41 | 9.81 | 15 | 4.29 | 56 | 7.29 | 47 | 11.27 | 33 | 9.43 | 80 | 10.43 |
| Black or African American | 21 | 5.02 | 12 | 3.43 | 33 | 4.30 | 31 | 7.43 | 27 | 7.71 | 58 | 7.56 |
| Native American or American Indian | 2 | 0.48 | 4 | 1.14 | 6 | 0.78 | 5 | 1.20 | 2 | 0.57 | 7 | 0.91 |
| Other | 10 | 2.39 | 6 | 1.71 | 16 | 2.08 | 10 | 2.40 | 10 | 2.86 | 20 | 2.61 |
| Prefer not to say | 3 | 0.72 | 3 | 0.86 | 6 | 0.78 | 2 | 0.48 | 3 | 0.86 | 5 | 0.65 |
| White or Caucasian | 341 | 81.58 | 310 | 88.57 | 651 | 84.77 | 322 | 77.22 | 275 | 78.57 | 597 | 77.84 |
| Total | 418 | 100 | 350 | 100 | 768 | 100 | 417 | 100 | 350 | 100 | 767 | 100 |
