# Supplementary material for: Depression, anxiety, and happiness in dog owners and potential dog owners during the COVID-19 pandemic in the United States
Source: PLoS One. 2021 Dec 15;16(12):e0260676. doi: 10.1371/journal.pone.0260676 (PMC8673598; doi:10.1371/journal.pone.0260676)
Supplement: S16 Table — (DOCX) [file pone.0260676.s016.docx]

**S16 Table. Hispanic origin or descent.**

|  | Dog owners | | | | | | Potential dog owners | | | | | |
| --- | --- | --- | --- | --- | --- | --- | --- | --- | --- | --- | --- | --- |
|  | 11/2020 | | 02/2021 | | Final sample | | 11/2020 | | 02/2021 | | Final sample | |
|  | n | % | n | % | n | % | n | % | n | % | n | % |
| Yes | 31 | 7.42 | 22 | 6.29 | 53 | 6.90 | 27 | 6.47 | 23 | 6.57 | 50 | 6.52 |
| No | 387 | 92.58 | 328 | 93.71 | 715 | 93.10 | 390 | 93.53 | 327 | 93.43 | 717 | 93.48 |
| Total | 418 | 100 | 350 | 100 | 768 | 100 | 417 | 100 | 350 | 100 | 767 | 100 |
