# Supplementary material for: Depression, anxiety, and happiness in dog owners and potential dog owners during the COVID-19 pandemic in the United States
Source: PLoS One. 2021 Dec 15;16(12):e0260676. doi: 10.1371/journal.pone.0260676 (PMC8673598; doi:10.1371/journal.pone.0260676)
Supplement: S17 Table — (DOCX) [file pone.0260676.s017.docx]

**S17 Table. Perceived impact of Covid-19 on finances.**

Fifty-five percent of dog owners (54.68%) and potential dog owners (54.89%) indicated that the pandemic had little to no effect on their finances. Twenty-six percent of dog owners (25.65%) and potential dog owners (25.81%) indicated that it had a somewhat negative impact on their finances. Twenty percent of dog owners (19.66%) and nineteen percent (19.29%) of potential dog owners reported that the pandemic had a very to extremely negative effect on their finances.

| **On my finances/financially** | Dog owners | | | | | | Potential dog owners | | | | | |
| --- | --- | --- | --- | --- | --- | --- | --- | --- | --- | --- | --- | --- |
|  | 11/2020 | | 02/2021 | | Final sample | | 11/2020 | | 02/2021 | | Final sample | |
|  | n | % | n | % | n | % | n | % | n | % | n | % |
| Extremely negative effect | 33 | 7.89 | 29 | 8.29 | 62 | 8.07 | 34 | 8.15 | 28 | 8.00 | 62 | 8.08 |
| Very negative effect | 49 | 11.72 | 40 | 11.43 | 89 | 11.59 | 47 | 11.27 | 39 | 11.14 | 86 | 11.21 |
| Somewhat negative effect | 112 | 26.79 | 85 | 24.29 | 197 | 25.65 | 113 | 27.10 | 85 | 24.29 | 198 | 25.81 |
| Little negative effect | 116 | 27.75 | 98 | 28.00 | 214 | 27.86 | 111 | 26.62 | 101 | 28.86 | 212 | 27.64 |
| No negative effect at all | 108 | 25.84 | 98 | 28.00 | 206 | 26.82 | 112 | 26.86 | 97 | 27.71 | 209 | 27.25 |
| Total | 418 | 99.99* | 350 | 100.01* | 768 | 99.99* | 417 | 100 | 350 | 100 | 767 | 99.99* |

* Total not equal to 100% due to rounding error.
