# Supplementary material for: Depression, anxiety, and happiness in dog owners and potential dog owners during the COVID-19 pandemic in the United States
Source: PLoS One. 2021 Dec 15;16(12):e0260676. doi: 10.1371/journal.pone.0260676 (PMC8673598; doi:10.1371/journal.pone.0260676)
Supplement: S18 Table — (DOCX) [file pone.0260676.s018.docx]

**S18 Table. Perceived impact of Covid-19 on emotions.**

Thirty-three percent of dog owners (33.21%) and thirty-two percent (31.56%) of potential dog owners indicated that the pandemic had little to no effect on their emotions. Thirty-five percent of dog owners (35.29%) and potential dog owners (35.20%) indicated that it had a somewhat negative impact on their emotions. Thirty-two percent of dog owners (31.51%) and thirty-three percent (33.24%) of potential dog owners reported that the pandemic had a very to extremely negative effect on their emotions.

| **On my emotions/emotionally** | Dog owners | | | | | | Potential dog owners | | | | | |
| --- | --- | --- | --- | --- | --- | --- | --- | --- | --- | --- | --- | --- |
|  | 11/2020 | | 02/2021 | | Final sample | | 11/2020 | | 02/2021 | | Final sample | |
|  | n | % | n | % | n | % | n | % | n | % | n | % |
| extremely negative effect | 38 | 9.09 | 31 | 8.86 | 69 | 8.98 | 36 | 8.63 | 34 | 9.71 | 70 | 9.13 |
| very negative effect | 95 | 22.73 | 78 | 22.29 | 173 | 22.53 | 93 | 22.30 | 79 | 22.57 | 172 | 22.43 |
| somewhat negative effect | 148 | 35.41 | 123 | 35.14 | 271 | 35.29 | 149 | 35.73 | 121 | 34.57 | 270 | 35.20 |
| little negative effect | 91 | 21.77 | 79 | 22.57 | 170 | 22.14 | 93 | 22.30 | 77 | 22.00 | 170 | 22.16 |
| no negative effect at all | 46 | 11.00 | 39 | 11.14 | 85 | 11.07 | 46 | 11.03 | 39 | 11.14 | 85 | 11.08 |
| Total | 418 | 100 | 350 | 100 | 768 | 100.01* | 417 | 99.99* | 350 | 99.99* | 767 | 100 |

* Total not equal to 100% due to rounding error.
