# Supplementary material for: Depression, anxiety, and happiness in dog owners and potential dog owners during the COVID-19 pandemic in the United States
Source: PLoS One. 2021 Dec 15;16(12):e0260676. doi: 10.1371/journal.pone.0260676 (PMC8673598; doi:10.1371/journal.pone.0260676)
Supplement: S19 Table — (DOCX) [file pone.0260676.s019.docx]

**S19 Table. Perceived impact of Covid-19 on health.**

Sixty-six percent of dog owners (66.28%) and of potential dog owners (66.49%) indicated that the pandemic had little to no effect on their health. Twenty-four percent of dog owners (24.09%) and of potential dog owners (23.99%) indicated that it had a somewhat negative impact on their health. Ten percent of dog owners (9.63%) and of potential dog owners (9.52%) reported that the pandemic had a very to extremely negative effect on their health.

| **On my health/health-wise** | Dog owners | | | | | | Potential dog owners | | | | | |
| --- | --- | --- | --- | --- | --- | --- | --- | --- | --- | --- | --- | --- |
|  | 11/2020 | | 02/2021 | | Final sample | | 11/2020 | | 02/2021 | | Final sample | |
|  | n | % | n | % | n | % | n | % | n | % | n | % |
| extremely negative effect | 11 | 2.63 | 10 | 2.86 | 21 | 2.73 | 11 | 2.64 | 9 | 2.57 | 20 | 2.61 |
| very negative effect | 26 | 6.22 | 27 | 7.71 | 53 | 6.90 | 27 | 6.48 | 26 | 7.43 | 53 | 6.91 |
| somewhat negative effect | 96 | 22.97 | 89 | 25.43 | 185 | 24.09 | 96 | 23.02 | 88 | 25.14 | 184 | 23.99 |
| little negative effect | 152 | 36.36 | 115 | 32.86 | 267 | 34.77 | 148 | 35.49 | 117 | 33.43 | 265 | 34.55 |
| no negative effect at all | 133 | 31.82 | 109 | 31.14 | 242 | 31.51 | 135 | 32.37 | 110 | 31.43 | 245 | 31.94 |
| Total | 418 | 100 | 350 | 100 | 768 | 100 | 417 | 100 | 350 | 100 | 767 | 100 |
