# Supplementary material for: Depression, anxiety, and happiness in dog owners and potential dog owners during the COVID-19 pandemic in the United States
Source: PLoS One. 2021 Dec 15;16(12):e0260676. doi: 10.1371/journal.pone.0260676 (PMC8673598; doi:10.1371/journal.pone.0260676)
Supplement: S20 Table — (DOCX) [file pone.0260676.s020.docx]

**S20 Table. Perceived impact of Covid-19 on lifestyle.**

Twenty-eight percent of dog owners (28.12%) and twenty-nine percent (28.55%) of potential dog owners indicated that the pandemic had little to no effect on their lifestyle. Forty percent of dog owners (39.84%) and of potential dog owners (40.16%) indicated that it had a somewhat negative impact on their lifestyle. Thirty-two percent of dog owners (32.05%) and thirty-one percent (31.29%) of potential dog owners reported that the pandemic had a very to extremely negative effect on their lifestyle.

| **On my lifestyle** | Dog owners | | | | | | Potential dog owners | | | | | |
| --- | --- | --- | --- | --- | --- | --- | --- | --- | --- | --- | --- | --- |
|  | November 2020 | | February 2021 | | Final sample | | November 2020 | | February 2021 | | Final sample | |
|  | n | % | n | % | n | % | n | % | n | % | n | % |
| extremely negative effect | 37 | 8.85 | 35 | 10.00 | 72 | 9.38 | 39 | 9.35 | 34 | 9.71 | 73 | 9.52 |
| very negative effect | 95 | 22.73 | 79 | 22.57 | 174 | 22.67 | 82 | 19.66 | 85 | 24.29 | 167 | 21.77 |
| somewhat negative effect | 170 | 40.67 | 136 | 38.86 | 306 | 39.84 | 172 | 41.25 | 136 | 38.86 | 308 | 40.16 |
| little negative effect | 86 | 20.57 | 72 | 20.57 | 158 | 20.57 | 92 | 22.06 | 69 | 19.71 | 161 | 20.99 |
| no negative effect at all | 30 | 7.18 | 28 | 8.00 | 58 | 7.55 | 32 | 7.67 | 26 | 7.43 | 58 | 7.56 |
| Total | 418 | 100 | 350 | 100 | 768 | 100.01* | 417 | 99.99* | 350 | 100 | 767 | 100 |

* Total not equal to 100% due to rounding error.
