# Supplementary material for: Depression, anxiety, and happiness in dog owners and potential dog owners during the COVID-19 pandemic in the United States
Source: PLoS One. 2021 Dec 15;16(12):e0260676. doi: 10.1371/journal.pone.0260676 (PMC8673598; doi:10.1371/journal.pone.0260676)
Supplement: S21 Table — (DOCX) [file pone.0260676.s021.docx]

**S21 Table. Pet Attitude Scale descriptive statistics.**

|  | Dog owners | | | Potential dog owners | | |
| --- | --- | --- | --- | --- | --- | --- |
|  | 11/2020 | 02/2021 | Final sample | 11/2020 | 02/2021 | Final sample |
| Minimum | 36 | 51 | 36 | 50 | 66 | 50 |
| Maximum | 126 | 126 | 126 | 126 | 126 | 126 |
| Mean | 110.57 | 109.17 | 109.93 | 105.34 | 106.66 | 105.94 |
| Standard deviation | 12.64 | 14.42 | 13.49 | 13.17 | 13.14 | 13.16 |
