# Supplementary material for: Depression, anxiety, and happiness in dog owners and potential dog owners during the COVID-19 pandemic in the United States
Source: PLoS One. 2021 Dec 15;16(12):e0260676. doi: 10.1371/journal.pone.0260676 (PMC8673598; doi:10.1371/journal.pone.0260676)
Supplement: S22 Table — (DOCX) [file pone.0260676.s022.docx]

**S22 Table. Miller-Rada Commitment to Pets Scale descriptive statistics.**

|  | Dog owners | | | Potential dog owners | | |
| --- | --- | --- | --- | --- | --- | --- |
|  | 11/2020 | 02/2021 | Final sample | 11/2020 | 02/2021 | Final sample |
| Minimum | 18 | 14 | 14 | 13 | 10 | 10 |
| Maximum | 50 | 50 | 50 | 50 | 50 | 50 |
| Mean | 44.18 | 43.73 | 43.97 | 41.35 | 41.66 | 41.49 |
| Standard deviation | 7.06 | 7.47 | 7.25 | 7.52 | 7.83 | 7.66 |
