# Supplementary material for: Depression, anxiety, and happiness in dog owners and potential dog owners during the COVID-19 pandemic in the United States
Source: PLoS One. 2021 Dec 15;16(12):e0260676. doi: 10.1371/journal.pone.0260676 (PMC8673598; doi:10.1371/journal.pone.0260676)
Supplement: S23 Table — (DOCX) [file pone.0260676.s023.docx]

**S23 Table. Multidimensional Scale of Perceived Social Support descriptive statistics.**

|  | Dog owners | | | Potential dog owners | | |
| --- | --- | --- | --- | --- | --- | --- |
|  | 11/2020 | 02/2021 | Final sample | 11/2020 | 02/2021 | Final sample |
| Minimum | 1 | 1.25 | 1 | 1 | 1 | 1 |
| Maximum | 7 | 7 | 7 | 7 | 7 | 7 |
| Mean | 5.44 | 5.52 | 5.48 | 5.29 | 5.41 | 5.34 |
| Standard deviation | 1.21 | 1.25 | 1.23 | 1.22 | 1.34 | 1.28 |
