# Supplementary material for: Depression, anxiety, and happiness in dog owners and potential dog owners during the COVID-19 pandemic in the United States
Source: PLoS One. 2021 Dec 15;16(12):e0260676. doi: 10.1371/journal.pone.0260676 (PMC8673598; doi:10.1371/journal.pone.0260676)
Supplement: S24 Table — (DOCX) [file pone.0260676.s024.docx]

**S24 Table. Center for Epidemiologic Studies Depression Scale-Revised descriptive statistics.**

|  | Dog owners | | | Potential dog owners | | |
| --- | --- | --- | --- | --- | --- | --- |
|  | 11/2020 | 02/2021 | Final sample | 11/2020 | 02/2021 | Final sample |
| Minimum | 0 | 0 | 0 | 0 | 0 | 0 |
| Maximum | 67 | 69 | 69 | 76 | 68 | 76 |
| Mean | 12.86 | 11.86 | 12.41 | 14.56 | 13.45 | 14.06 |
| Standard deviation | 14.51 | 13.94 | 14.25 | 14.89 | 14.80 | 14.86 |
