# Supplementary material for: Depression, anxiety, and happiness in dog owners and potential dog owners during the COVID-19 pandemic in the United States
Source: PLoS One. 2021 Dec 15;16(12):e0260676. doi: 10.1371/journal.pone.0260676 (PMC8673598; doi:10.1371/journal.pone.0260676)
Supplement: S25 Table — (DOCX) [file pone.0260676.s025.docx]

**S25 Table. Generalized Anxiety Disorder Scale descriptive statistics.**

|  | Dog owners | | | Potential dog owners | | |
| --- | --- | --- | --- | --- | --- | --- |
|  | 11/2020 | 02/2021 | Final sample | 11/2020 | 02/2021 | Final sample |
| Minimum | 0 | 0 | 0 | 0 | 0 | 0 |
| Maximum | 21 | 21 | 21 | 21 | 21 | 21 |
| Mean | 4.61 | 4.21 | 4.43 | 4.87 | 4.76 | 4.82 |
| Standard deviation | 5.11 | 4.96 | 5.04 | 5.35 | 5.19 | 5.27 |

S3 Table 6. Oxford Happiness Scale.

|  | Dog owners | | | Potential dog owners | | |
| --- | --- | --- | --- | --- | --- | --- |
|  | 11/2020 | 02/2021 | Final sample | 11/2020 | 02/2021 | Final sample |
| Minimum | 1.14 | 1.55 | 1.14 | 1.35 | 1.35 | 1.35 |
| Maximum | 5.86 | 6.0 | 6.0 | 5.72 | 5.90 | 5.90 |
| Mean | 4.04 | 4.07 | 4.05 | 3.95 | 4.04 | 3.99 |
| Standard deviation | 0.90 | 0.88 | 0.89 | 0.87 | 0.94 | 0.91 |
