# Supplementary material for: Depression, anxiety, and happiness in dog owners and potential dog owners during the COVID-19 pandemic in the United States
Source: PLoS One. 2021 Dec 15;16(12):e0260676. doi: 10.1371/journal.pone.0260676 (PMC8673598; doi:10.1371/journal.pone.0260676)
Supplement: S27 Table — (DOCX) [file pone.0260676.s027.docx]

**S27 Table. Correlations between attitude towards pets and depression, anxiety, and happiness scores.**

| Kendall correlation | overall | dog owner | potential dog owners |
| --- | --- | --- | --- |
| Attitude / Depression | -0.04 | -0.07 | 0.00 |
| Attitude/ Anxiety | -0.04 | -0.05 | -0.01 |
| Attitude/ Happiness | 0.14 | 0.18 | 0.08 |
