# Supplementary material for: Depression, anxiety, and happiness in dog owners and potential dog owners during the COVID-19 pandemic in the United States
Source: PLoS One. 2021 Dec 15;16(12):e0260676. doi: 10.1371/journal.pone.0260676 (PMC8673598; doi:10.1371/journal.pone.0260676)
Supplement: S28 Table — (DOCX) [file pone.0260676.s028.docx]

**S27 Table. Correlations between commitment to pets and depression, anxiety, and happiness scores.**

| Kendall correlation | overall | dog owner | potential dog owners |
| --- | --- | --- | --- |
| Commitment / Depression | -0.04 | -0.05 | -0.02 |
| Commitment / Anxiety | -0.03 | -0.04 | -0.00 |
| Commitment / Happiness | 0.09 | 0.12 | 0.06 |
